# Prevalence of Antibodies to Crimean-Congo Hemorrhagic Fever Virus in Ruminants, Nigeria, 2015

**DOI:** 10.3201/eid2604.190354

**Published:** 2020-04

**Authors:** Daniel Oluwayelu, Babak Afrough, Adebowale Adebiyi, Anitha Varghese, Park Eun-Sil, Shuetsu Fukushi, Tomoki Yoshikawa, Masayuki Saijo, Eric Neumann, Shigeru Morikawa, Roger Hewson, Oyewale Tomori

**Affiliations:** University of Ibadan, Ibadan, Nigeria (D. Oluwayelu, A. Adebiyi);; Public Health England, Salisbury, UK (B. Afrough, A. Varghese, R. Hewson);; National Institute of Infectious Diseases, Shinjuku, Tokyo, Japan (P. Eun-Sil, S. Fukushi, T. Yoshikawa, M. Saijo, S. Morikawa);; Massey University, Palmerston North, New Zealand (E. Neumann);; Nigerian Academy of Science, Yaba, Nigeria (O. Tomori)

**Keywords:** prevalence, antibodies, Crimean-Congo hemorrhagic fever virus, CCHFV, viruses, Crimean-Congo hemorrhagic fever, CCHF, ruminants, zoonoses, Nigeria

## Abstract

Crimean-Congo hemorrhagic fever virus (CCHFV) is a highly transmissible human pathogen. Infection is often misdiagnosed, in part because of poor availability of data in disease-endemic areas. We sampled 150 apparently healthy ruminants throughout Nigeria for virus seropositivity and detected virus-specific IgG in cattle (24%) and goats (2%), highlighting the need for further investigations.

Crimean-Congo hemorrhagic fever (CCHF) is a fatal, zoonotic, tickborne viral infection endemic to Africa, the Middle East, Asia, and Europe. The CCHF virus (CCHFV) is primarily maintained in nature in *Hyalomma* ticks (*1*). Although faunae of different species become infected and sustain a transient viremia, overt disease in animals is not easily recognized. Most CCHF cases occur in humans involved in the livestock industry, including slaughterhouse workers and veterinarians (*2*). This occupational hazard is exacerbated by the asymptomatic infection that CCHFV establishes in animals.

Studies conducted over 3 decades suggest circulation of CCHFV in animals and ticks in Nigeria (*3*,*4*). However, this circulation might not reflect current virus activity in the livestock population of this country. Furthermore, the transborder movement of cattle, goats, and sheep into Nigeria from neighboring countries, such as Burkina Faso and Niger, where CCHF cases have occurred (*5*,*6*), could result in importation of CCHFV-infected animals and ticks. The role of domestic ruminants in CCHFV zoonosis in Nigeria has not been extensively investigated despite a report of CCHF epidemic in the United Arab Emirates caused by imported livestock and ticks from Somalia and Nigeria (*7*). This finding underpins the urgent need for assessment of the current status of CCHF among ruminants in Nigeria. We report the results of a pilot study conducted to investigate the prevalence of CCHFV antibodies in cattle, goats, and sheep from different vegetation zones of Nigeria.

## The Study

**Figure 1:**
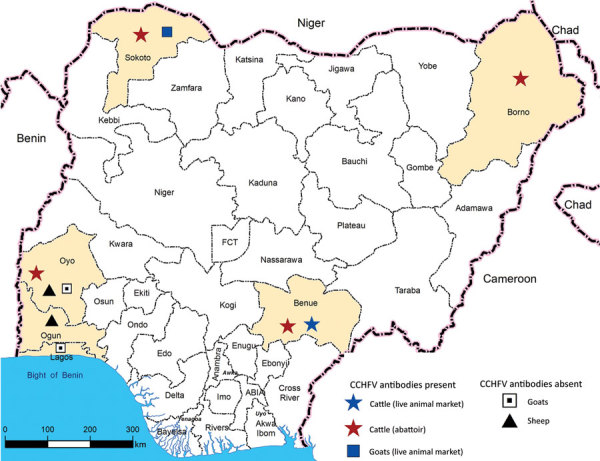
Sample collection sites and distribution of CCHFV antibody-positive and -negative samples in Nigeria. Yellow shading indicates states from which samples were collected. CCHFV, Crimean-Congo hemorrhagic fever virus; FCT, Federal Capital Territory.

During January–May 2015, we collected 150 serum samples (50 each from cattle, goats, and sheep) from live animal markets, abattoirs, and privately owned farms in different states in Nigeria. We collected cattle serum samples from the states of Sokoto (northwest, Sudan savannah zone), Borno (northeast, Sudan savannah), Benue (northcentral, Guinea savannah) and Oyo (southwest, rainforest) (Figure 1). Sokoto and Borno States have potential for transborder spread of diseases, such as CCHF, because they have borders with Niger, Chad, and Cameroon, 3 countries from which cattle are continually transported into Nigeria. We collected goat serum samples from the states of Lagos (southwest, rainforest) Oyo, and Sokoto and sheep serum samples from the states of Ogun (southwest, rainforest) and Oyo (Figure 1).

All animals sampled were adults and apparently healthy at the time of sampling. We heat-inactivated serum samples (56°C, 30 min) and transported them on dry ice to Public Health England (Salisbury, UK) where they were stored at −20°C until used. Approval for this study was provided by the University of Ibadan/University College Hospital Ethics Committee (NHRFC/05/01/2008a).

We performed an ELISA by using recombinant CCHFV nucleoprotein (NP) as described (*8*). We coated half of the ELISA plate wells with purified recombinant CCHFV NP and half with negative control antigen and incubated the plates at 4°C overnight. After washing, we incubated the plates at 37°C with blocking solution for 1 h, 4-fold diluted (1:100–1:6400) test serum samples for 1 h, horseradish peroxidase–labeled anti-bovine or anti-goat IgG for 1 h, and 2,2'-azino-bis(3-ethylbenzothiazoline-6-sulphonic acid) substrate at 25°C for 30 min. Each incubation was followed by a washing step.

We determined sensitivity and specificity of the ELISA initially by creating receiver operating characteristic (ROC) and 2-graph ROC curves with Stat Flex version 5 software (*9*). We estimated an optimal cutoff point by comparing a range of sensitivity and specificity values for a range of cutoff values.

To confirm true-positive ELISA results, we performed an indirect fluorescent antibody test (IFAT) with recombinant CCHFV NP antigens as described (*10*). We reacted serially diluted (1:20–1:80) serum samples with recombinant antigens on multiwell slides, incubated at 37°C for 1 h, and washed. We then incubated slides with Protein G, Alexa Fluor 488 conjugate (https://www.thermofisher.com) at 37°C for 1 h, washed the slides, and examined them by using fluorescent microscopy.

**Figure 2:**
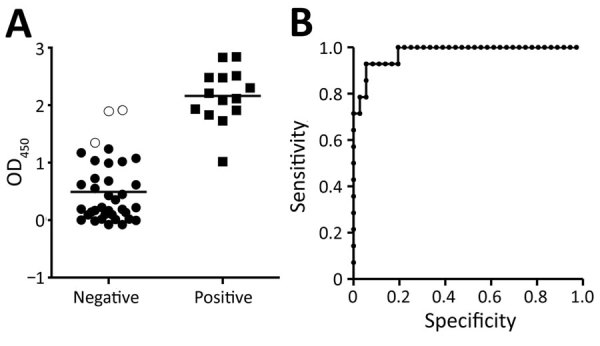
Prevalence of antibodies to Crimean-Congo hemorrhagic fever virus (CCHFV) in ruminants, Nigeria, 2015. A) Distribution of ELISA OD values for serum samples from cattle according to indirect fluorescent antibody test (IFAT) results, Nigeria. The OD at 405 nm for each serum sample at a dilution of 1:400 in the ELISA was plotted against serum samples from CCHFV IFAT antibody-positive and antibody-negative cattle. Three antibody-negative samples (open circles) were above the cutoff OD value in the ELISA and thus considered to be false positive in the ELISA. Other antibody-negative samples (solid circles) were also ELISA negative. Horizontal bars indicate mean OD values. Solid squares indicate IFAT-positive serum samples. B) Receiver operating characteristic (ROC) analysis of an IgG-ELISA specific for nucleoprotein of CCHFV. Area under the ROC curve was 0.9762, which indicates that the test has a good probability of distinguishing between CCHFV antibody–positive and –negative cattle. OD, optical density.

We obtained optical density (OD) values of serum samples tested with the ELISA at a 1:400 dilution and compared the distribution of OD values for IFAT-positive and IFAT-negative samples (Figure 2, panel A). Areas under the 2-graph ROC curve were 0.976 and 1.000 for cattle (Figure 2, panel B), which indicated that the test had a good probability of distinguishing between CCHFV antibody–positive and –negative animals. Because only 1 goat sample was positive by IFAT, the cutoff OD value for goat was not determined by 2-graph ROC analysis.

Seroprevalence rates obtained by the CCHFV IgG-ELISA were 24.0% for cattle, 2.0% for goats, and 0% for sheep (Table). Fourteen (28.0%) of the 50 cattle serum samples were positive for CCHFV NP antibodies by IFAT: 3 (15%) from an abattoir in Oyo State, 4 (40%) from a live animal market in Borno State, 1 (10%) from an abattoir in Sokoto State, and 6 (60%) from an abattoir and a live animal market in Benue State. Only 1 (2.0%) of the 50 goat serum samples was positive by IFAT; this positive sample was obtained from an adult female at a live animal market in Sokoto State. We did not test sheep serum samples by IFAT. We found 96.0% concordance between ELISA and IFAT results for cattle.

**Table:** CCHFV IgG ELISA antibody prevalence among cattle, goats, and sheep in Nigeria, 2015*

State	Cattle				Goats				Sheep		
	No. tested	No. (%) positive			No. tested	No. (%) positive			No. tested	No. (%) positive	
		ELISA	IFAT			ELISA	IFAT			ELISA	IFAT
Oyo	20	3 (15.0)	3 (15.0)		13	0	0		30	0	NT
Lagos	NS	–	–		2	0	0		NS	–	–
Ogun	NS	–	–		NS	–	–		20	0	NT
Benue	10	5	6		NS	–	–		NS	–	–
Sokoto	10	1	1		35	1 (2.9)	1 (2.9)		NS	–	–
Borno	10	3	4		NS	–	–		NS	–	–
Total	50	12 (24.0)	14 (28.0)		50	1 (2.0)	1 (2.0)		50	0	–

*CCHFV, Crimean-Congo hemorrhagic fever virus; IFAT, indirect fluorescent antibody test; NS, no samples, NT, not tested; –, no results.

## Conclusions

Serologic and virologic evidence of CCHFV in humans has been reported in Nigeria (*11*). However, to our knowledge, CCHFV presence among domestic animals in the country has not been documented since the studies of Causey et al. (*3*) and Umoh et al. (*4*) conducted >3 decades ago. Detection of CCHFV antibodies in domestic animals is useful because it provides evidence of circulating virus, identifies the location of CCHFV foci, and highlights a potential and increased risk for human infection (*12*). Thus, our findings provide valuable serologic proof of the continued occurrence of CCHFV infection among domestic ruminants in Nigeria because there is no vaccine currently available against this disease (*12*).

We obtained higher CCHFV antibody prevalence rates for cattle, indicating that they were more exposed to the virus than goats and sheep. This finding suggests that in Nigeria, cattle might play a major role in the maintenance, circulation, and epidemiology of CCHFV, an observation that corroborates earlier reports that CCHFV infection appeared to occur most frequently in larger mammals such as cattle, which are the preferred hosts of adult *Hyalomma* ticks (*13*).

Although we found 96% concordance between ELISA and IFAT in this study, the IFAT detected 2 additional positive samples from cattle. This discrepancy could be related to differences in serum starting dilutions used in the 2 tests: 1:100 for the ELISA and 1:20 for the IFAT. Nevertheless, the higher CCHFV seropositivity obtained in the Sudan savannah (Sokoto and Borno states) and Guinea savannah (Benue State) zones is consistent with a report from Senegal (*14*), which found that CCHFV transmission was most intense in the northern, drier, and sparsely vegetated Sahelian ecozone than in the southern, more humid sub-Guinean (rainforest) zone.

Although *Hyalomma* ticks are the principal transmitters of CCHF in nature, transmission can also occur in their absence (i.e., person-to-person or by infected animals in abattoirs). Moreover, the prevalence of CCHFV antibodies in livestock and humans coincides with the distribution of these arthropods (*15*). Thus, detection of seropositive animals in this study provides good evidence of circulation of the virus among cattle and goats in Nigeria, making them a public health risk. Therefore, our findings highlight the need to fully investigate the transmission dynamics between tick reservoirs and virus-amplifying ruminants across Nigeria to engender improved understanding of virus–host relationships, the characterization of circulating CCHFV, improved diagnostics and monitoring systems, and better disease control.
